# Early-Life Intervention Using Exogenous Fecal Microbiota Alleviates Gut Injury and Reduce Inflammation Caused by Weaning Stress in Piglets

**DOI:** 10.3389/fmicb.2021.671683

**Published:** 2021-06-10

**Authors:** Xin Ma, Yuchen Zhang, Tingting Xu, Mengqi Qian, Zhiren Yang, Xiuan Zhan, Xinyan Han

**Affiliations:** ^1^The Key Laboratory of Molecular Animal Nutrition, Ministry of Education, College of Animal Science, Zhejiang University, Hangzhou, China; ^2^Key Laboratory of Animal Nutrition and Feed Science in East China, Ministry of Agriculture, College of Animal Science, Zhejiang University, Hangzhou, China; ^3^Hainan Institute of Zhejiang University, Hainan, China

**Keywords:** fecal microbiota transplantation, weaning stress, intestinal microbiota, diarrhea, piglets

## Abstract

Fecal microbiota transplantation (FMT) could shape the structure of intestinal microbiota in animals. This study was conducted to explore the changes that happen in the structure and function of microbiota caused by weaning stress, and whether early-life FMT could alleviate weaning stress through modifying intestinal microbiota in weaned piglets. Diarrheal (D) and healthy (H) weaned piglets were observed, and in the same farm, a total of nine litters newborn piglets were randomly allocated to three groups: sucking normally (S), weaned at 21 d (W), and early-life FMT + weaned at 21 d (FW). The results demonstrated that differences of fecal microbiota existed in group D and H. Early-life FMT significantly decreased diarrhea incidence of weaned piglets. Intestinal morphology and integrity were improved in the FW group. Both ZO-1 and occludin (tight junction proteins) of jejunum were greatly enhanced, while the zonulin expression was significantly down-regulated through early-life FMT. The expression of IL-6 and TNF-α (intestinal mucosal inflammatory cytokines) were down-regulated, while IL-10 (anti-inflammatory cytokines) was up-regulated by early-life FMT. In addition, early-life FMT increased the variety of the intestinal microbial population and the relative amounts of some beneficial bacteria such as *Spirochaetes*, *Akkermansia*, and *Alistipes*. Functional alteration of the intestinal microbiota revealed that lipid biosynthesis and aminoacyl-tRNA biosynthesis were enriched in the FW group. These findings suggested that alteration of the microbiota network caused by weaning stress induced diarrhea, and early-life FMT alleviated weaning stress in piglets, which was characterized by decreased diarrhea incidence, improved intestinal morphology, reduced intestinal inflammation, and modified intestinal bacterial composition and function.

## Introduction

Weaning acts as a pivotal part in a pig’s life. Early weaning has been widely used in pig production as it helps to shorten the pig slaughter cycle and improve the reproductive performance of sows (Campbell et al., 2013). Under the condition of the modern large-scale intensive pig production, weaning is usually carried out at 3–4 weeks of age. However, weaning stress is generated due to separation from the mother, dietary change from highly digestible liquid milk, handling, transport, and alteration of physical environments during the period of weaning (Sutherland et al., 2014). Weaning-induced stress will lead to loss of appetite, post-weaning diarrhea, growth retardation, intestinal inflammation, and un-balanced gut microbiota (Pié et al., 2004). There is plenty of evidence that gut microbes can help prevent diarrhea (Youmans et al., 2015; Arroyo et al., 2020). In-feed antibiotics are often used to prevent and relieve weaning stress-induced diarrhea. However, antibiotics have been strictly managed or even banned in the modern animal husbandry due to increased antibiotic resistance and residues in foods (Allen et al., 2014). In China, in-feed antibiotics were banned from July, 2020. Thus, there is a pressing need for exploiting non-antibiotic alternative strategies to help alleviate the weaning stress.

The intestinal epithelial barrier, which includes intestinal epithelial cells and intercellular junctions, especially the intestinal tight junctions (TJs), plays a central role in defending against the invasion of microbiota, toxins, and antigens from the exogenous environment (Rescigno, 2011). In various gastrointestinal diseases such as diarrhea and inflammatory bowel disease (IBD), the damage of intestinal epithelial barrier function has been confirmed (Laukoetter et al., 2008). The intestinal epithelial barrier is not a static physical barrier but is constantly interacting with the intestinal microbiota. Accumulating studies indicate that intestinal microbiota affect maintenance of intestinal epithelial barrier through modulating the immune system (Takiishi et al., 2017). Weaning stress can cause impairment of the intestinal epithelial barrier and alteration of intestinal microbiota (Li et al., 2018). However, therapies for alleviating weaning stress through modulation of the intestinal microbiota are scarce.

Fecal microbiota transplantation (FMT), which refers to transferring the whole microbial environment from healthy individual stool into the recipient’s gut, is aimed at normalizing or modifying intestinal microbiota composition and function (Khoruts and Sadowsky, 2016). The first recordation on this technique was from Ge Hong. He was a Chinese physician in the Jin Dynasty who took advantage of the FMT method to remedy severe diarrhea. Recently, FMT has been revealed to contribute to the treatment of IBD (Paramsothy et al., 2017), and FMT is a better selection for Clostridium difficile infection (CDI) patients who do not respond to antibiotics (Weingarden et al., 2013; Fischer et al., 2015). Although FMT appears to be effective in clinical applications, knowledge about the effect of FMT in domestic animals is still limited.

In our previous research, we found that Duroc × Landrace × Yorkshire crossbred newborn pigs that received exogenous fecal microbiota from local adult Jinhua pigs exhibited modified intestinal microbiota structure and intestinal development (Hu et al., 2018). FMT treatment could alleviate LPS-induced intestinal epithelial injury (Geng et al., 2018). In view of this beneficial effect, we hypothesized that early-life FMT could alleviate weaning stress through modulating microbiota structure and improve intestinal function in piglets. In this study, we probed into the structural and functional changes of gut microbiota between diarrheal weaned piglets and healthy weaned piglets, the protective roles of FMT in piglets undergoing weaning stress and the impacts of early-life FMT on gastrointestinal microbiota and intestinal health in weaned piglets. Microbial functions were further analyzed to illustrate how FMT influenced the microbiota and improved intestinal homeostasis. As far as we know, this is the first experiment in which FMT technology is applied to alleviate weaning stress. This study may provide new insights in alleviating weaning stress in mammals through microbiota intervention.

## Results

### Diversity and Structure of Fecal Microbiota in Diarrhea Piglets

**FIGURE 1:**
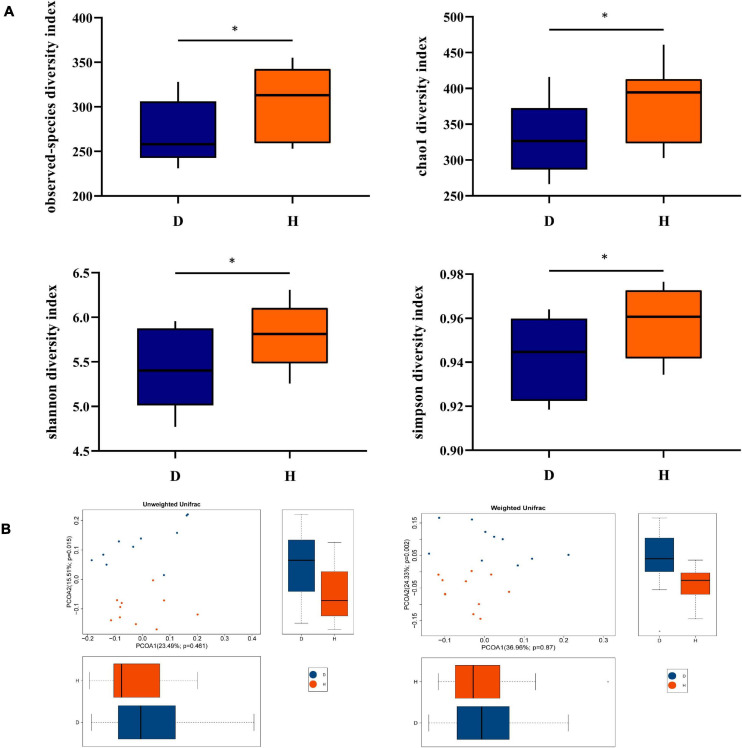
Diversity analysis of fecal microbiota between groups D and H. **(A)** Alpha diversity is the index of species variety in a single sample, including the observed species, chao1, shannon, and simpson. **(B)** Principal Coordinates Analysis (PCoA) was used to further demonstrate the differences in species diversity between samples, it can reveal the magnitude of the differences between samples. PCoA analysis results of species diversity between samples, if two samples are close to each other, it means that the species composition of these two samples is similar (D, Diarrhea; H, Health). *difference between the two groups is significant(*p* < 0.05).

Alpha diversity indices were calculated for examining the variety of the fecal microbiota between diarrheal and healthy piglets. As Figure 1A shows, observed species, Chao1, Shannon, and Simpson indices were lower in D pigs, indicating diarrhea decreased the richness of the fecal microbiota. These indicated that diarrhea significantly decreased the richness and diversity of microbiota. To analyze the diversity between individuals, PCoA was conducted, which suggested that the composition of fecal microbiota had differences between two groups (Figure 1B).

**FIGURE 2:**
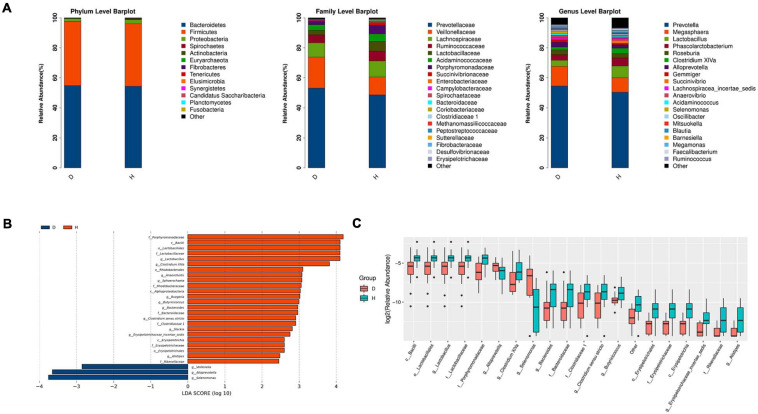
Microbiota structure analysis of two groups. **(A)** According to the results of species annotation, the corresponding histogram of crop species profiling in phyla, family, and genus can be used to intuitively check the species with higher relative abundance and their proportion in different classification levels of each group. **(B)** LDA Effect Size (LEfSe analysis): LEfSe used linear discriminant analysis (LDA) to estimate the impact of the abundance of each component (species) on the difference effect and to identify the community or species that had a significant impact on the sample division (The LDA threshold is 2). **(C)** Boxplot result of genus level differential species. (D, Diarrhea; H, Health).

The relative abundance of different phyla, families, and genera across two groups were shown in Figure 2A. At the phylum level, Bacteroidetes was the most abundant in group D (54.83%) and group H (54.36%), followed by Firmicutes (42.78, 41.67%, respectively). It was worth mentioning that Fusobacteria was found only in the group H but was undetected in the group D. At the family level, Prevotellaceae was the most abundant in group D (53.20%) and group H (48.70%), followed by Veillonellaceae (20.64, 11.91%, respectively) and Lachnospiraceae (9.45, 10.60%, respectively). At the genus level, *Prevotella* was the most abundant in both two groups. These results also suggested that the microbiome diversity and structure were changed in group D.

LEfSe analysis further indicated that diarrhea decreased the diversity of microbiota. The results showed that there were 3 and 22 discriminating taxa in the groups D and H, respectively. As shown in Figure 2B, Lactobacillaceae, Rhodobacteraceae and Erysipelotrichaceae played a crucial role in the group H. *Clostridium XIVa*, *Anaerofustis*, *Sphaerochaeta*, *Ruegeria*, *Erysipelotrichaceae incertae sedis*, and *Alistipes* were important in group H. Furthermore, the results in Figure 2C showed that there were significant increases in the relative abundance of *Lactobacillus*, *Bacteroides*, *Butyricicoccus*, *Slackia*, and *Clostridium sensu stricto*. However, the abundance of *Veillonella*, *Alloprevotella*, and *Selenomonas* was raised in the group *D. overall*, diarrheal piglets had the different fecal microbiota construction from healthy piglets.

**FIGURE 3:**
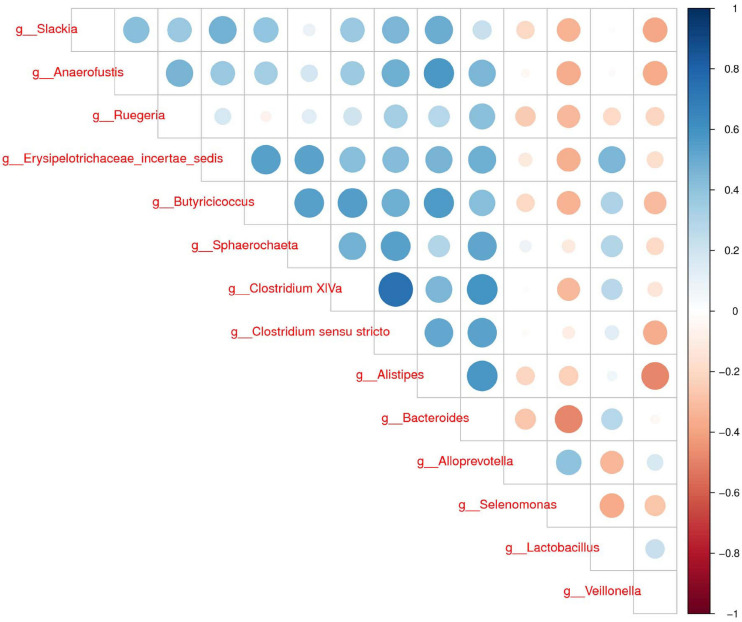
Correlation analysis of fecal microbiota. Spearman correlation heat map among species. At all taxonomic levels, the correlation coefficients between species with differences in abundance Top 30 are positively correlated in blue and negatively correlated in red on the right. The darker the color, the stronger the correlation between species.

To further determine the relationships of different microbes between two groups, correlation analysis of fecal microbiota was performed by calculating Spearman’s correlation coefficients among all genera. Figure 3 revealed that the higher interactions of gut microbiota were found in the group H, and the two most relevant genera were Clostridium XIVa and *Clostridium sensu stricto* (0.75). *Veillonella* was correlated negatively with *Alistipes*, *Slackia*, and *Anaerofustis*. Our results suggested less connection in the microbial community caused by diarrhea.

**FIGURE 4:**
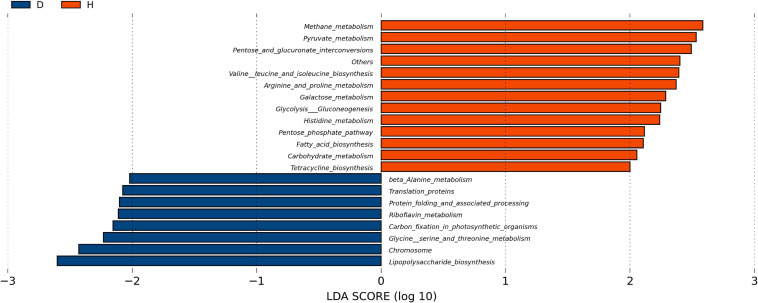
Function analysis of fecal microbiota. PICRUSt (phylogenetic investigation of Communities by reconstruction of unobserved States) studies the phylogenetic investigation of community system evolution through the recessive state. The abscisic is the log value obtained by LDA of KEGG pathway with significant roles in two groups, and different colors indicated that the KEGG pathway was enriched in different groups of samples. (D, Diarrhea; H, Health).

In order to explore the functional changes between groups D and H, the microbiota in each fecal sample was analyzed by the PICRUSt method. These results were presented in Figure 4. The predicted proportions of the genes involved in lipopolysaccharide biosynthesis, protein translation, riboflavin and β-alanine metabolism were significantly increased in the group D. The relative abundance of pyruvate metabolism, tetracycline biosynthesis, arginine, histidine metabolism, fatty acid biosynthesis, pentose phosphate pathway and other genes in group D were predicted to be lower.

### Weight Gain and Diarrhea Incidence of Piglets

**FIGURE 5:**
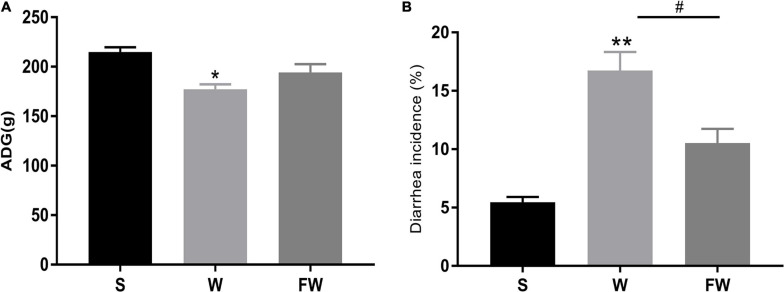
ADG **(A)** and diarrhea incidence **(B)** of group S, W, and FW. The symbol “*” represented that the W and FWs group were compared with the S group, the symbol “#” represented that the FW group was compared with the W group. Data were shown as mean ± SEM (*p < 0.05, **p < 0.01, and ^#^*p* < 0.05) (S, Sucking; W, Weaned; FW: FMT+Weaned). (1) ADG was calculated as weight gain (final body weight-initial body weight) divided by the number of treatment days. (2) The incidence of diarrhea (%) was calculated as the total number of diarrheal piglets during the period divided by the total number of piglets multiplies duration of the trial.

As shown in Figure 5A, there was a significant decrease in the ADG of the W group compared with S (*P* < 0.05). There were no significant differences between the W group and FW group, but the latter had an increasing trend in ADG (0.05 < *P* < 0.1). As shown in Figure 5B, compared with S group, diarrhea incidence was significantly increased in the W group (*P* < 0.01). And the diarrhea incidence in the FW group was decreased compared to the W group (*P* < 0.05). In addition, there were no differences in ADG and diarrhea incidence between the S group and FW group (*P* > 0.05).

### Intestinal Barrier Function

**FIGURE 6:**
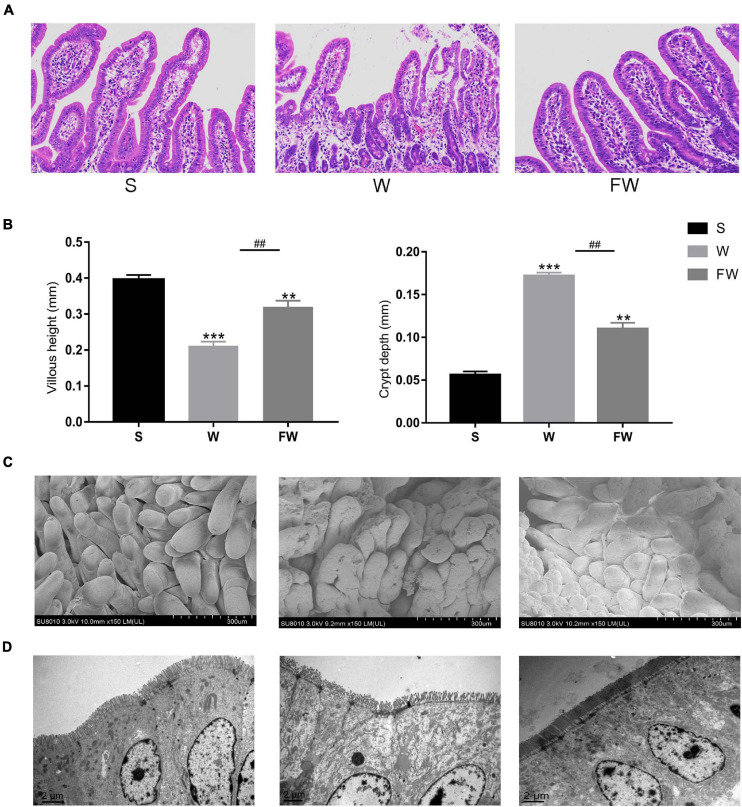
Jejunum sections were used to analyze intestinal morphology. **(A)** Stained with H&E (bars, 200 μ m). **(B)** The villus height (mm) and crypt depth (mm) were measured. Data were represented as mean ± SEM for the three different experiments (*p < 0.05, **p < 0.01, ***p < 0.001, ^#^*p* < 0.05, ^##^*p* < 0.01, and ^###^*p* < 0.001). **(C)** SEM images (150×). **(D)** TEM images (8,000×). (S, Sucking; W, Weaned; FW, FMT+Weaned).

As shown in Figures 6A,B, the villous height of jejunum was significantly increased, while the crypt depth was significantly decreased in FW group compared with W group. This result was observed through SEM at 150× magnification (Figure 6C). The smoother and more intact villi were observed in FW group than that in W group, and the surface damage to villi in the W group was alleviated through early-life FMT (Figure 6C). The quantity and height of microvilli increased in the FW group of jejunum compared to the W group (Figure 6D). These results suggested that early-life FMT improved intestinal morphology and integrity in weaned piglets.

**FIGURE 7:**
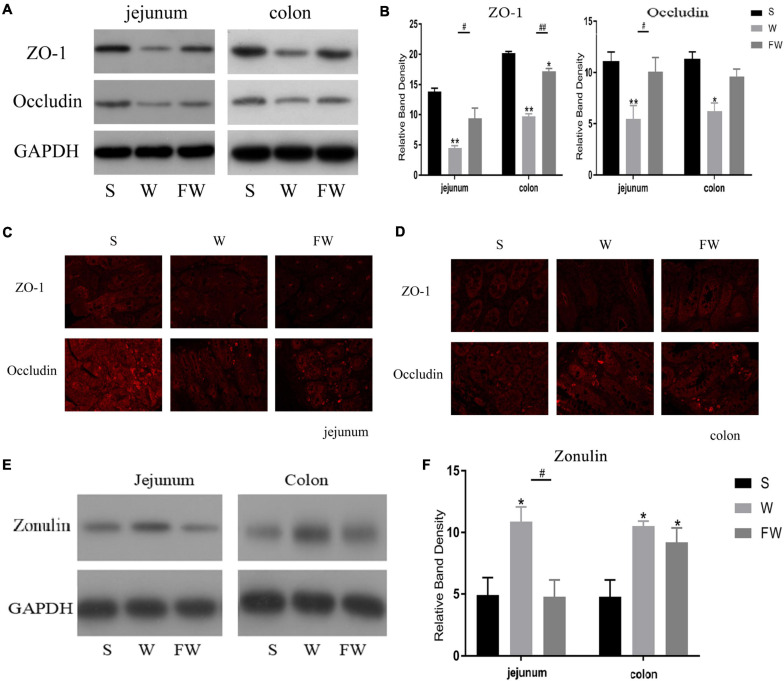
Intestinal mucosal tight junction proteins expression of three groups. **(A,B)** Western blot measurements of the expression levels of ZO-1 and occludin in jejunum and colon. **(C,D)** The distributions of ZO-1 and occludin in jejunum and colon were determined by immunohistochemical staining (150×). **(E,F)** Western blot measurements of the expression levels of zonulin in jejunum and colon. The symbol “*” represented that the W and FW groups were compared with the S group, the symbol “#” represented that the FW group was compared with the W group. Data are shown as mean ± SEM for the three different experiments (*p < 0.05, **p < 0.01, ^#^*p* < 0.05, and ^##^*p* < 0.01) (S, Sucking; W, Weaned; FW, FMT+Weaned).

Western blot was performed to analyze the expression of ZO-1, occludin and zonulin in jejunal and colonic mucosae, and immunofluorescence image was used to verify the distribution of ZO-1 and occludin. Expression of jejunum mucosal ZO-1 and occludin in the FW group was higher than that in the W group (*P* < 0.05), while there were no differences in the FW group and S group (*P* > 0.05; Figures 7A,B). In colonic mucosa, the expression of ZO-1 decreased in the W group (*P* < 0.05). However, there were no difference in the expression of occludin between the W and FW group (*P* > 0.05; Figures 7A,B). These results could be observed in immunofluorescence analysis (Figures 7C,D). In addition, expression of zonulin in jejunum was higher in the W group (*P* < 0.05), however there were no differences in the expression of zonulin in the colon between the W and FW group (*P* > 0.05; Figures 7E,F). These results indicated that early-life FMT enhanced the intestinal epithelial physical barrier.

### Expression of Intestinal Mucosal Inflammatory Cytokines

**FIGURE 8:**
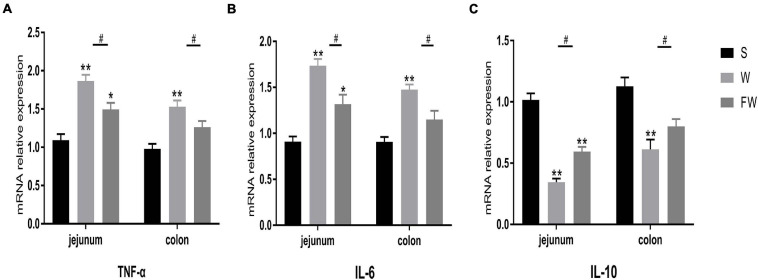
Intestinal mucosal cytokines level of three groups. The relative mRNA expression of proinflammatory cytokines IL-6 **(A)**, TNF-α **(B)**, and anti-inflammatory IL-10 **(C)** were determined by qRT-PCR. The symbol “*” represented that the W and FW groups were compared with the S group, the symbol “#” represented that the FW group was compared with the W group. Data were shown as mean ± SEM for the three different experiments (*p < 0.05, **p < 0.01, ***p < 0.001, and ^#^*p* < 0.05) (S, Sucking; W, Weaned; FW: FMT+Weaned).

Inflammatory cytokines IL-6 and TNF-α, anti-inflammatory cytokines IL-10 expression were measured to evaluate intestinal inflammation. The results showed that mRNA expression of IL-6 and TNF-α was increased (Figures 8A,B), while IL-10 was decreased in jejunum and colon after weaning (Figure 8C). Early-life FMT treatment resulted in lower mRNA expression of IL-6, TNF-α and higher mRNA expression of IL-10.

### Richness and Biodiversity of Intestinal Microbiota

**FIGURE 9:**
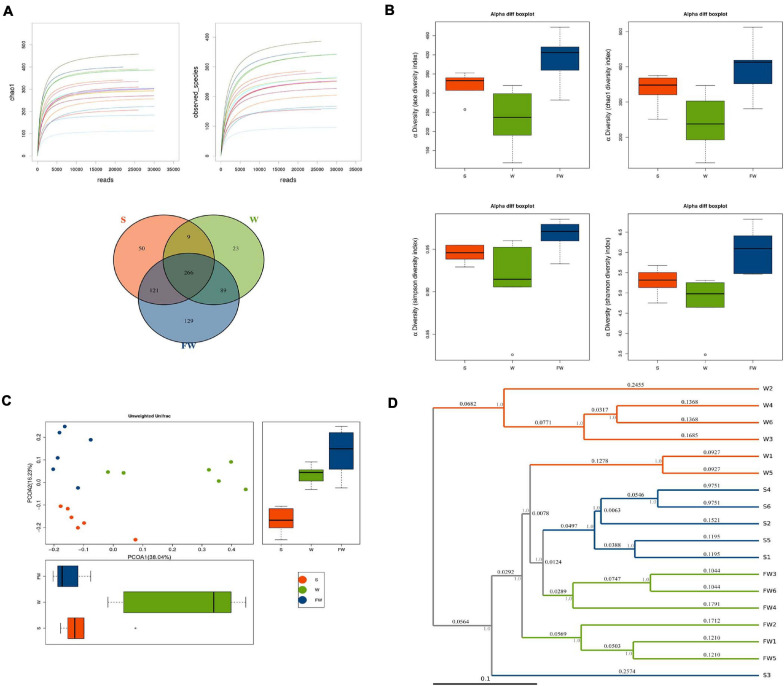
Richness and biodiversity of Intestinal Microbiota. **(A)** Rarefaction curves determined at the 97% similarity level and Venn diagram of OTUs in the three groups. **(B)** α-diversity indexes of bacterial community. **(C)** Scatter plots obtained from PCoA based on the unweighted UniFrac. **(D)** UPGMA hierarchical clustering analysis based on the unweighted UniFrac (S, Sucking; W, Weaned; FW, FMT+Weaned).

In order to explore whether FMT could alleviate the impact of weaning stress on intestinal microbiota, specimens were analyzed by sequencing the bacterial 16S rDNA V3-V4 region. After removing the low-quality sequences, an average of 35,672 high quality sequences per sample (*n* = 6) were subjected to the following analysis. The rarefaction curve indicates that the sampling is sufficient (Figure 9A). Further, the overlap of OTUs revealed that 266 OTUs coexisted in all three groups. A further 275 OTUs were presented in both the S and W groups, 355 in the W and FW groups, and 387 in the S and FW groups (Figure 9A).

Figure 9B showed that the alpha diversity among three groups, as measured by ACE, Chao1, Shannon, and Simpson indices. These indices showed that early-life FMT significantly increased the richness and diversity of microbiota (Supplementary Table 3). PCoA analysis revealed that gut microbiota in the W group separated from the baseline structure, and the FW group did not return to the level of the S group (Figure 9C). The system clustering tree revealed a significant difference existed in the three groups. Compared with the W group, the level of the FW group was closer to that of the S group (Figure 9D).

### Overall Structure of Intestinal Microbiota

**FIGURE 10:**
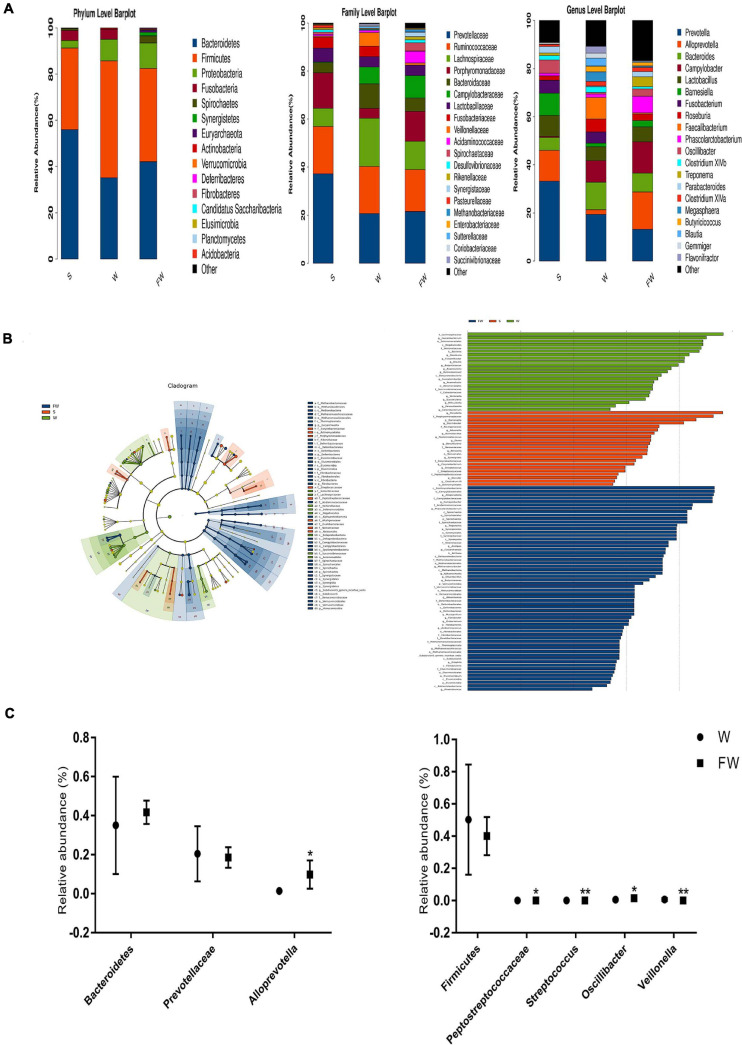
Intestinal microbial community structure of three groups. **(A)** Relative abundance of microbial community in the colon at phylum, family, and genus level. **(B)** Cladogram and LDA value distribution histogram based on LEfSe analysis showed significant differences of the microbial community in the three groups. **(C)** Differences of the relative abundance of Bacteroidetes, Prevotellaceae, *Alloprevotella*, Firmicutes, Peptostreptococcaceae, *Stretococcus*, *Oscillibacter*, and *Veillonella* between the W and FW group (S, Sucking; W, Weaned; FW, FMT+Weaned). *p < 0.05 and **p < 0.01.

As shown in Figure 10A, a total of 15 phyla were identified across three groups. Bacteroidetes was the most abundant phylum in the S group (55.75%) and FW group (41.70%), followed by Firmicutes (35.09, 40.03%, respectively) and Proteobacteria (3.28, 10.85%, respectively). In the W group, Firmicutes (50.24%) was the most predominant phylum, followed by Bacteroidetes (35.01%) and Proteobacteria (9.31%) (Supplementary Table 4). In addition, Fibrobacteres, Elusimicrobia, and Acidobacteria were found only in the FW group but undetected in the S and W groups. Candidatus and Planctomycetes were found in the S and FW group but not in the W group. These results suggested that the microbial structure was changed after early-FMT treatment. Furthermore, Prevotellaceae, Ruminococcaceae, Lachnospiraceae, Bacteroidaceae, and Porphyromonadaceae were the predominant bacterial families in colonic microbiota, collectively accounting for more than 70% of the bacterial communities within colon contents (Supplementary Table 7). At genus level, *Prevotella*, *Bacteroides*, *Campylobacter*, *Alloprevotella*, and *Lactoba-cillus* were the predominant genera (Supplementary Table 8). Detailed information about the relative abundance of bacteria in the class and order level was presented in Supplementary Figures 1,2 and Supplementary Tables 5, 6.

To determine the bacteria which were responsible for driving the FW group differences, we performed LEfSe analysis in the W and FW groups. For taxa with LDA scores greater than two in at least one group, we summarized the relative abundance across the two groups. A histogram of LDA scores was plotted to identify statistically significant biomarkers and to reveal the dominant microorganisms in the groups. The results showed that 12 taxa and 53 taxa were enriched in the W and FW groups, respectively. Among them, *Faecalibacterium*, *Veillonellaceae*, *Eubacteriaceae*, and *Flavonifractor* played an important role in the W group. *Alloprevotella*, *Porphyromonadaceae*, *Acidaminococcaceae*, and *Spirochaete* were enriched in the FW group (Figure 10B). An evolutionary clustering analysis diagram was generated based on the LDA score to identify important microflora using taxonomy. As shown in Figure 10C, the abundances of Archaea including Euryarchaeota phyla were increased in the FW group. And Streptococcaceae, Eubacteriacea, Betaproteobacteria, and Veillonellaceae were the major different microbiota in the W group. *Spirochaetes*, *Synergistetes*, *Verrucomicrobia*, *Deferribacteres*, and *Fibrobacteres* were identified as novel superior microbiota in the FW group.

**FIGURE 11:**
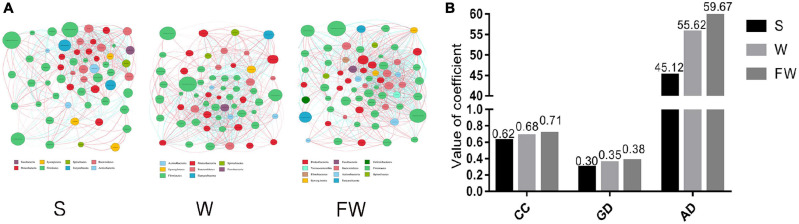
Network analysis of microbial community. **(A)** Microbial co-occurrence network analysis in three groups based on the calculation of Spearman’s correlation coefficients. The nodes represented different genus and their size represented the average relative abundance of the genus. The thickness of the connection between the nodes is positively correlated with the absolute value of the correlation coefficient of species interaction. The red lines indicated positive correlations and blue lines indicated negative correlations. **(B)** Typical coefficients derived from network analysis in three groups. CC, Clustering coefficient; GD, graph density; AD, Average degree (S, Sucking; W, Weaned; FW, FMT+Weaned).

The network analysis of gut microbiota was performed using Spearman’s correlation coefficients among all genera to further identify the relationships among different microbes in three groups. The absolute value of coefficient was set as more than 0.6 and a co-occurrence network was visualized graph viz -2.38.0. As shown in Figure 11, the higher interactions of gut microbiota were found in the FMT group, indicated by higher cluster coefficient (CC) (0.71), graph density (GD) (0.38) and average degree (AD) (59.67). The results indicated that the correlations of gut microbiota were enhanced through early-life FMT.

### Analysis of Intestinal Microbial Function

**FIGURE 12:**
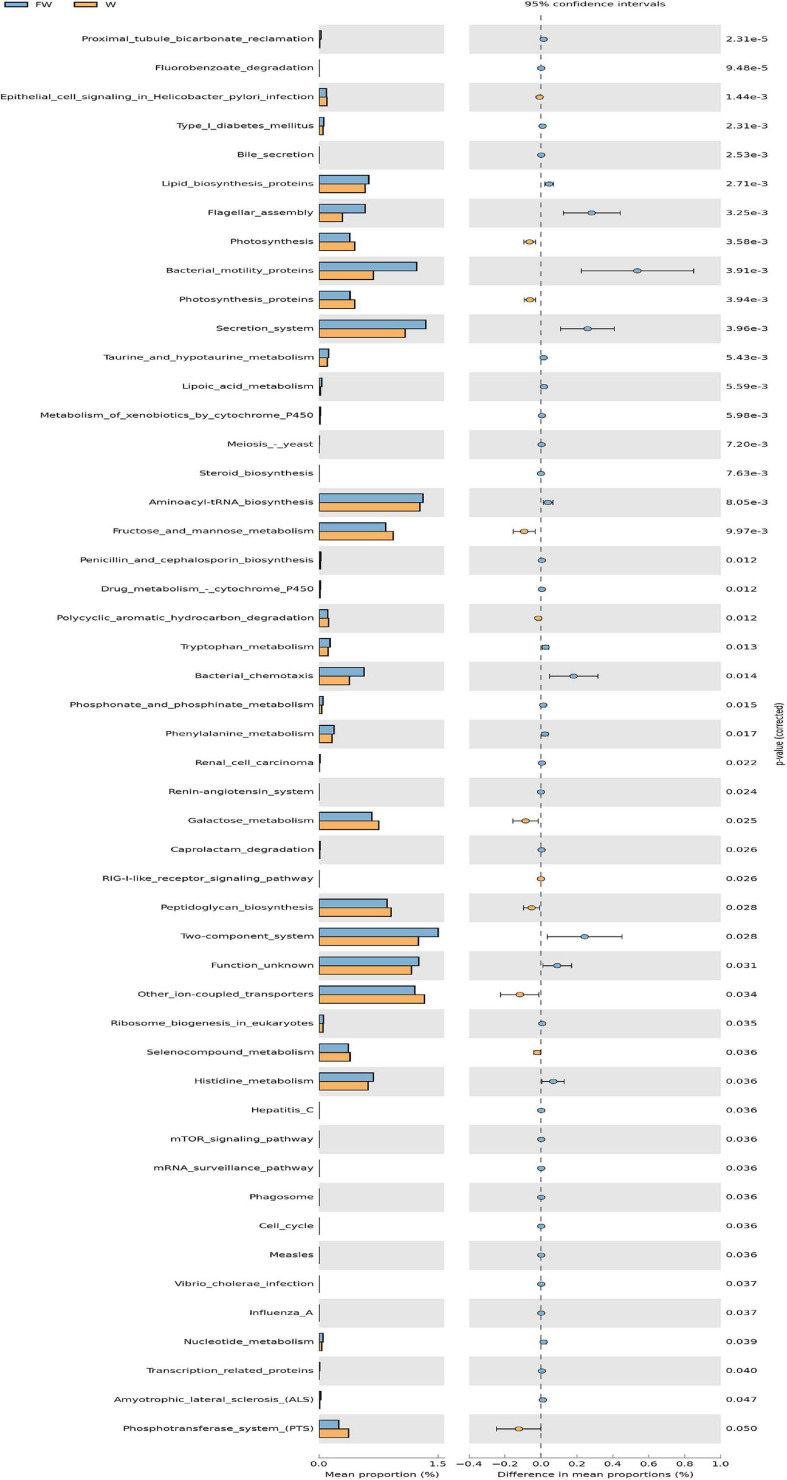
The comparison of predicted microbial function between the W and FW groups. STAMP analysis was applied to identify the significant differences in the predictive functions at the third level of KEGG pathways between two groups. The *P*-values were shown at right (W, Weaned; FW, FMT+Weaned).

In order to investigate the functional profiles between the W and FW groups, PICRUSt was used to predict the functions of bacterial communities based on the 16S rDNA sequencing data. As shown in Figure 12, the predicted relative abundances of the genes involved in lipid biosynthesis, aminoacyl-tRNA biosynthesis, histidine metabolism, and nucleotide metabolism were significantly increased in the FW group. Meanwhile, the predicted proportions of the genes for carbohydrate metabolism including fructose and mannose metabolism, galactose metabolism were significantly decreased in the W group.

## Discussion

The intestinal microbiota plays an important role in intestinal homeostasis. When something such as porcine epidemic diarrhea virus induces disorders of the intestinal microbiota, diarrhea happens (Huang et al., 2019). The diversity of microbiota is an indicator which is used to evaluate microbial composition. Reduced microbial alpha diversity in response to diarrhea is a common observation in diverse animal species including captive musk deer (Sun et al., 2020). The results showed that group D had lower observed species, Chao1, Shannon, and Simpson indices, indicating that α-diversity of fecal microbiota was decreased in diarrhea piglets. PCoA analysis also showed that a significant difference existed in two groups, which suggested a low similarity in the microbiome between group D and group H.

The LEfSe analysis showed that some genera such as *Veillonella*, *Alloprevotella*, and *Selenomonas* increased in group D. A significant increase of *Veillonella* was related to the ecological imbalance of intestinal microbiota in porcine epidemic diarrhea virus (PEDV) infected piglets (Wang et al., 2020). *Alloprevotella* was linked to diarrhea, the number of them increased when diarrhea score decreased (Tap et al., 2017). To go further, the elevation of lipopolysaccharide (LPS) biosynthesis in our functional prediction results may be associated with the increase in the abundance of these potentially harmful bacteria. And LPS biosynthesis plays a major role in bacterial pathogenesis (Wang et al., 2016). A previous study suggested a crucial role of the genus *Clostridium XIVa* in the intestinal barrier (Stokes, 2017). Our research also found that *Clostridium XIVa* was a differential genus between two groups. On the other hand, the amount of *Clostridium XIVa* was negatively correlated with *Alloprevotella* and *Selenomonas*, which indicated that diarrhea caused alteration in the fecal microbiota network.

Intestinal morphology is crucial to nutrient absorption. Weaning stress gives rise to significant changing of intestinal morphology and function in mammals, such as villus atrophy, crypt hyperplasia, decreased digestibility, and impaired intestinal barrier integrity and immune function (Oloughlin et al., 1991; Farhadi et al., 2003). The present result confirmed these adverse changes in the W group including damaged intestinal villi and increased crypt depth in the jejunum. While the increased villus height and decreased crypt depth were found in the FW group, which indicated that the capacity of nutrient absorption in intestine was enhanced (Ulluwishewa et al., 2011). This may explain the higher weight gain of piglets in the FW group. In addition, decreased diarrhea incidence may also contribute to the weight gain of early-life FMT treated piglets.

The intestinal epithelial barrier is responsible for preventing the invasion of harmful substances such as microorganisms, toxins, and LPS (Fasano, 2011). Damaged intestinal barrier would cause many gastrointestinal diseases, such as diarrhea and IBD. Tight junctions seal the gaps between epithelial cells, thus preventing the penetration of intestinal bacteria and other antigens next to the cells, and is therefore essential for the integrity of the intestinal barrier (Stevens et al., 2017). ZO-1 and occludin are known as the most considerable components of TJs in intestinal epithelial cells. Zonulin is a biomarker of gut epithelium tight junction barrier integrity, when the microbiota is disordered, it releases more (Mennigen et al., 2009; Wang et al., 2017), and zonulin could reversibly and rapidly regulate the TJs and change the intestinal permeability. Decreased expression of TJ proteins could increase the permeability of the epithelial barrier, and exacerbate inflammation (Haines et al., 2016). In the present study, we observed that the expressions of ZO-1 and occludin were decreased in the W group, while their expressions were higher in the FW group. The expression of zonulin was decreased in the FW group compared to the W group. These data suggested that early-life FMT was of benefit to the disrupted intestine epithelial barrier function triggered by weaning stress.

Cytokines are important mediators in the modification of immune and inflammatory responses. Studies have linked weaning to an up-regulation of expression of inflammatory cytokines, which are also involved in mediating the intestinal epithelial barrier dysfunction (Farhadi et al., 2003; Hooper and Macpherson, 2010; Kim et al., 2012). Consistent with previous studies, intestinal mucosal inflammatory cytokine IL-6 and TNF-α were typically increased, anti-inflammatory cytokine IL-10 was decreased in the intestine of piglets after weaning (Houghteling and Walker, 2015). In this study the expression of IL-6 and TNF-α was decreased, the expression of IL-10 was increased in FW group. These results indicated that intestinal inflammation and epithelial barrier dysfunction triggered by weaning stress was alleviated in piglets following early-life FMT.

In human and animal intestines, there are considerable and diverse microorganisms, supporting gut growth, pathogens resistance, and immune modulation (Frese et al., 2015). Moreover, the early establishment of the infant intestinal microbiota lays the foundation for the adult microbiome and had a long-term impact on host health (Gresse et al., 2017). Early weaned piglets were susceptible to pathogens due to weaning stress and immature gastrointestinal tract (Houghteling and Walker, 2015). Increasing studies showed that there was an abrupt taxonomic and functional shift in the intestinal microbiota of weaned piglets (Bäumler and Sperandio, 2016; Tian et al., 2016). The diminution in gut microbial diversity at weaning allows pathogenic organisms to access glycans in the mucus layer (Hu et al., 2016). FMT, as a bacterial intervention technique, could regulate diversity and structure of gut microbiota in mice or piglet models (Shukla et al., 2015; Hu et al., 2018). In our present study, higher ACE, Chao1, Shannon, and Simpson indices were found in the FW group, suggesting that the α-diversity of gut microbiota was increased in piglets following early-life FMT. Analysis of beta diversity showed that a significant difference existed among three groups, with a smaller difference between the S group and FW group. The result indicated a higher similarity in composition between the S group and FW group.

Consistent with previous studies, Bacteroidetes, Firmicutes, and Proteobacteria were the most abundant phyla in the intestinal tract (Bonder et al., 2016). To identify the specific bacterial taxa associated with FMT, the LEfSe method was performed to compare the gut microbiota of W and FW groups. The results showed that some opportunistic pathogens such as Veillonellaceae, Streptococcaceae and Streptococcus increased in the W group. Veillonellaceae was associated with a pro-inflammatory response in IBD, IBS, and cirrhosis patients (Qi et al., 2005; De et al., 2012), and *Streptococcus* was reported to be dominated in the microbiota of people who developed gastric diseases (Unno et al., 2015). While some health-promoting bacterial species including Fibrobacteres, *Spirochaetes*, *Akkermansia*, *Alistipes*, and *Oscillibacter* increased in the FW group. The Fibrobacteres can efficiently decompose cellulose and degrade hard-to-degrade plant polysaccharides, which helps the body digest fiber and metabolize energy (Shkoporov et al., 2008). It has been observed that the body weight of pigs was associated with increases in the abundance of Spirillaceae (Downes et al., 2013). Similarly, the increased proportion of Spirochaetaceae in the FW group may be linked to the increased weight gain of weaned piglets. *Alistipes*, *Alloprevotella*, and *Oscillospira* could produce short chain fatty acids (SCFAs), which are the anions that are quickly absorbed by the colonic epithelial cells (Peng et al., 2009; Fukuda et al., 2011; Konikoff and Gophna, 2016). Growing evidence indicates that SCFA can protect the host against colonic diseases, enhance the gut barrier function and exhibit anti-inflammatory effects (Langille et al., 2013; Kumari and Kozyrskyj, 2017). In conclusion, early fecal microbiota intervention can promote the healthy and balanced development of intestinal microbiota of weaned piglets by promoting the positive relationship between beneficial microbiota and inhibiting the competitiveness and proportion of potentially harmful bacteria. Together with the development of gut microbiota through early-life FMT, the predicted microbial function was also changed. The results in this study indicated an enrichment of protein metabolism capacity of the gut microbiome in the FW group. The significantly increased relative abundances in the KEGG pathways including aminoacyl-tRNA bio-synthesis, histidine metabolism and nucleotide metabolism, suggests that the capacity of protein digestion and absorption was enhanced (Mangalam et al., 2013). In addition, carbohydrate metabolism including fructose and mannose metabolism and galactose metabolism was decreased in the W group.

Early-life FMT modified the structure and function of the bacterial community, which may account for improved intestinal morphology and intestinal homeostasis in weaned piglets, thus reducing the incidence of diarrhea caused by weaning stress. It is known that microbiota can produce some metabolites which contribute to intestinal barrier integrity, immune regulation, and inflammation (Han et al., 2014). Therefore, further study would aim at the metabolic changes through FMT-mediated gut microbiota.

## Materials and Methods

### Experimental Design and Animal Management

In this experiment, all methods were carried out in accordance with the Guide for the Care and Use of Laboratory Animals prepared by the Institutional Animal Care and Use Committee of Zhejiang University, and animals used in this study were authorized by the principles of the Zhejiang University Animal Care and Use Committee (No. 2012-0178). Weaning piglets with similar birth dates were observed. Seven days after being weaned, 10 piglets were randomly selected from different pens with diarrhea as the diarrhea group (D), and their feces samples were collected. At the same time, feces of 10 piglets which were not diarrheal (group H) were collected. Diarrhea in this experiment included mild diarrhea and severe diarrhea, referred to a previous study (Atarashi et al., 2013). In the same farm, a total of nine litters (9–11 piglets per litter) of DLY newborn piglets with similar birth dates and parity were used in this study. After being fed with colostrum, piglets were divided into three groups with three litters in each, i.e., (1) Sucking group (S); (2) Weaned group (W); (3) FMT + Weaned group (FW). In the FW group, fecal microbial suspension was taken orally, while piglets in the W group were orally inoculated with the same volume of sterile PBS. The fecal suspension from a healthy Jinhua pig was prepared as previously described (Hu et al., 2018). The dosage was 1.5 ml/piglet once every other day from the first day to 14 days of age. Piglets in the W group and the FW group were weaned at the age of 21 days, while piglets in the S group were suckling normally until 24 days. Piglets fed a corn/soybean-based diet and had free access to feed and water. All nutrients reached or exceeded NRC (2012) recommendations. The diet composition for early-weaned piglets is shown in Supplementary Table 1. At the start and end of the feeding trial, piglets were weighed individually and average daily gain (ADG) was calculated, and the number of piglets with diarrhea and its duration were recorded during all experiment.

### Sample Collection

At 24 days of age, six piglets with normal feces (Atarashi et al., 2013) were euthanized from each group and then for sample collection. The samples of colonic contents were immediately frozen in liquid nitrogen and then placed at −80°C. The samples of jejunum and colon tissues were collected from the approximately middle positions, then PBS was used to remove contents and then fixed in 10% formalin, 2.5% glutaraldehyde and 4% paraformaldehyde for further analysis. The mucosal samples from jejunum and colon were collected by scraping with a sterile glass microscope slide, rapidly frozen in liquid nitrogen and stored at -80°C for further analysis.

### DNA Extraction and 16S rDNA Gene Amplicon Sequencing

Total Microbial genomic DNA of feces and colon content was extracted using E.Z.N.A^®^ Stool DNA Kits (Omega Biotek, Norcross, United States) according to the instructions of the manufacturer. Bacterial universalprimers (341F: ACTCCTACGG-GAGGCAGCAG, 806R: GGACTACHVGGGTWTCTAAT) were used for the amplification of the V3–V4 region of the bacterial 16S rDNA gene and subsequent pyrosequencing of the PCR products. Amplicons were confirmed by electrophoresis on a 2% agarose gel, purified with the AxyPrep DNA kit (AXYGEN, Tewksbury, MA, United States), quantified by Qubit 2.0 Fluorometer (Thermo Fisher Scientific, Waltham, United States) to pool into even concentration. Amplicon libraries were sequenced on Illumina Miseq PE250 platform (Illumina, San Diego, United States).

### Sequencing Data Analysis

The paired end reading of 2 × 250 bp was obtained through the MiSeq platform. High-quality sequences were obtained using Pandaseq. Then removed tags of which length lower than 220 nt, average quality score lower than 20, and ambiguous bases more than 3. The data were dechimerized and clustered using Usearch software, and the OTU (operational taxon) was obtained using standard clustering with 97% similarity. The taxonomy-based analysis to the OTUs was performed by RDP algorithm using GreenGene database^1^. Rank sum test analysis was used for analyzing alpha diversity, which included calculation of the ace, Chao 1, Shannon, and Simpson indices, and venn, principal coordinate analysis (PCoA), and unweighted pair group method with arithmetic mean (UPGMA) were performed using QIIME for beta-diversity analysis. The dominant bacterial community difference between groups was detected using Linear discriminant analysis (LDA) effect size (LEfSe).

### Microbial Function Prediction

Phylogenetic Investigation of Communities by Reconstruction of Unobserved States (PICRUSt) was used to predict the gene family abundances of bacterial communities based on the 16S rDNA gene data and a database of reference genomes (Han et al., 2014). STAMP was used to detect the different Kyoto Encyclopedia of Genes and Genomes (KEGG) pathways and false discovery rate correction. Two-sided Welch’s *t*-test and Benjamini–Hochberg FDR correction were used in two-group analysis. Statistical analysis of colonic taxonomy was conducted for normal distribution by Student’s *t*-test. Differences were considered significant at *P* < 0.05.

### Intestinal Morphology Examination

The jejunum and colon fractions were taken out from 4% paraformaldehyde solution, and then washed and embedded them in paraffin. Next, these fractions were cut into approximately 5-μm thick pieces then stained with hematoxylin, followed by eosin (H&E). Image processing and analysis systems were used to determine morphological indices (Version 1, Leica Imaging Systems Ltd., Cambridge, United Kingdom) according to our previous description (Atarashi et al., 2013).

The fixed mid-jejunum samples were washed three times in phosphate buffer(0.1 M, pH7.0) and then fixed with 1% OsO4 for 1h. The jejunum specimens were then dehydrated in a graded series of ethanol (30, 50, 70, 80, 90, 95, and 100%) for 20min at each step and transferred into a mixture of alcohol and iso-amyl acetate (v:*v* = 1:1) for 30min and iso-amyl acetate for 1h. After being dehydrated with liquid CO2 by a critical point dryer (Hitachi Model HCP-2, Japan), the segments were coated with gold-palladium and visualized using a Scanning Electron Microscope (SEM, Philips Model TM-1000, Japan).

Microvilli were viewed through TEM, and jejunum tissue was fixed, dehydrated, and transferred into pure acetone for 20min. The specimen was placed in a mixture of absolute acetone (1:1 for 1 h and 1:3 for 3 h) and then transferred into final Spurr resin mixture overnight. Next, the tissue sections were placed in eppendorf contained Spurr resin and heated at 70°C for more than 9 h. The specimens were then sectioned in LEICA EM UC7 ultratome and sections were stained by uranyl acetate and alkaline lead citrate for 5–10 min, respectively, and observed in Hitachi Model H-7650 TEM.

### Intestinal Mucosal Cytokine mRNA Expression

The mRNA expression of IL-6, TNF-α, and IL-10 were carried out by real-time PCR. Total RNA was extracted from jejunal and colonic mucosae using the TRIzol^®^ Plus RNA Purification Kit (Invitrogen, United States). RNA was spectrophotometrically quantified and its integrity was examined by agarose gel electrophoresis. Reverse transcription using the SuperScript^TM^ III First-Strand Synthesis SuperMix for qRT-PCR was carried out following the manufacturer’s instructions. Quantitative analysis of PCR was carried out on a CFX384 real-time fluorescent quantitative PCR system using a Power SYBR Green PCR Master Mix (Applied Biosystems, 4367659). The primers referred were presented in Supplementary Table 2. Each sample was repeated three times and the relative expression level of each gene was analyzed statistically by 2^(−ΔΔ*ct*)^.

### Intestinal Mucosal Tight Junction Proteins Expression

The expressions of ZO-1, occludin, and zonulin in the jejunal and colonic mucosa were identified by western blot. Total protein was extracted with T-PER Tissue Protein Extraction Reagent (Thermo Pierce, 78510), followed by total protein quantification using the BCA quantification kit. Protein supernatant was separated by 10% SDS-PAGE and transferred onto a nitrocellulose membrane. The membrane was incubated with the appropriate primary antibodies overnight at 4°C after blocking with 5% skimmed milk powder. Then, Goat anti-Mouse IgG (H + L) or Goat anti-Rabbit IgG (H + L). Secondary antibodies were added and incubated at room temperature for 1 h. Finally, the signals were detected using an enzyme-linked enhanced chemiluminescence (ECL) DualVue WB Marker (GE, 34080) according to the manufacturer’s protocol. The following antibodies were used in the western blot assays: ZO-1 (ThermoFisher 61-7300), occludin (Biorbyt orb11181), zonulin (Abcam ab231976). Protein detection was performed using SuperSignal^®^ West Dura Extended Duration Substrate (Thermo Pierce, 34075) and Image J software was used for densitometric analysis. GADPH protein was used as a housekeeping protein to determine relative abundance of a target protein.

### Immunofluorescence Analysis

Immunofluorescence was used to study the distribution of tight junction protein ZO-1 and occludin in jejunal and colonic tissues. Briefly, the tissue sections were deparaffinized and rehydrated, then Ethylene Diamine Tetra Acetic Acid (EDTA) Antigen Retrieval Solution (pH = 8.0) (Servicebio, China) was submitted to antigen retrieval. Washing slides three times with PBS (pH 7.4) in a Rocker device, the tissue pieces were blocked in BSA for 30 min and incubated with rabbit ZO-1 antibody (Servicebio, GB11195) and occludin antibody (Abcam, ab216327) overnight at 4°C. Finally, pieces were stained for 10 min at room temperature in the dark using the 4’,6-diamidino-2-phenylindole (DAPI) solution (Servicebio, G1012). The digital images of intestinal morphology were picked up through the fluorescence microscope.

### Statistical Analysis

Statistical analysis was performed by SPSS 20.0 (Chicago, IL, United States). Student’s *t*-test was used to analyze the means of two groups. One-way analysis of variance (ANOVA) followed by Tukey’s test was used to analyze the means of three groups. Data were presented as mean ± SEM. A level of *P* < 0.05 was considered as statistically significant.

## Conclusion

In conclusion, diarrhea can impact on the feces microbiota of weaned piglets, especially on the variety, construction, and function. Early-life FMT alleviated weaning stress in piglets, including the decreased diarrhea incidence, improved intestinal morphology, as well as reduced intestinal inflammation. Additionally, the composition and function of intestinal microbiota were changed through early-life FMT. In this study, exogenous fecal microbiota transportation was first found to exert a positive effect on alleviating weaning stress in piglets, and thus stands for an important step in the characterization of the influence of weaning stress upon the microbiota and the consequential impacts upon host physiology.

## Data Availability Statement

The original contributions presented in the study are publicly available. This data can be found here: NCBI repository, https://www.ncbi.nlm.nih.gov/sra/PRJNA714441.

## Ethics Statement

The animal study reviewed and approved by the Institutional Animal Care and Use Committee of Zhejiang University.

## Author Contributions

XH and XZ designed the experiments. XM and YZ performed the experiments. YZ and TX analyzed the data. XM wrote the manuscript, which was edited by YZ, MQ, ZY, and XH. All authors read and approved the final manuscript.

## Conflict of Interest

The authors declare that the research was conducted in the absence of any commercial or financial relationships that could be construed as a potential conflict of interest.

## Footnotes

1 http://greengenes.lbl.gov
