# Supplementary material for: Early-Life Intervention Using Exogenous Fecal Microbiota Alleviates Gut Injury and Reduce Inflammation Caused by Weaning Stress in Piglets
Source: Front Microbiol. 2021 Jun 10;12:671683. doi: 10.3389/fmicb.2021.671683 (PMC8222923; doi:10.3389/fmicb.2021.671683)
Supplement: Supplementary file 1 [file Data_Sheet_1.docx]

Supplementary Material

# Supplementary Figures and Tables

## Supplementary Figures


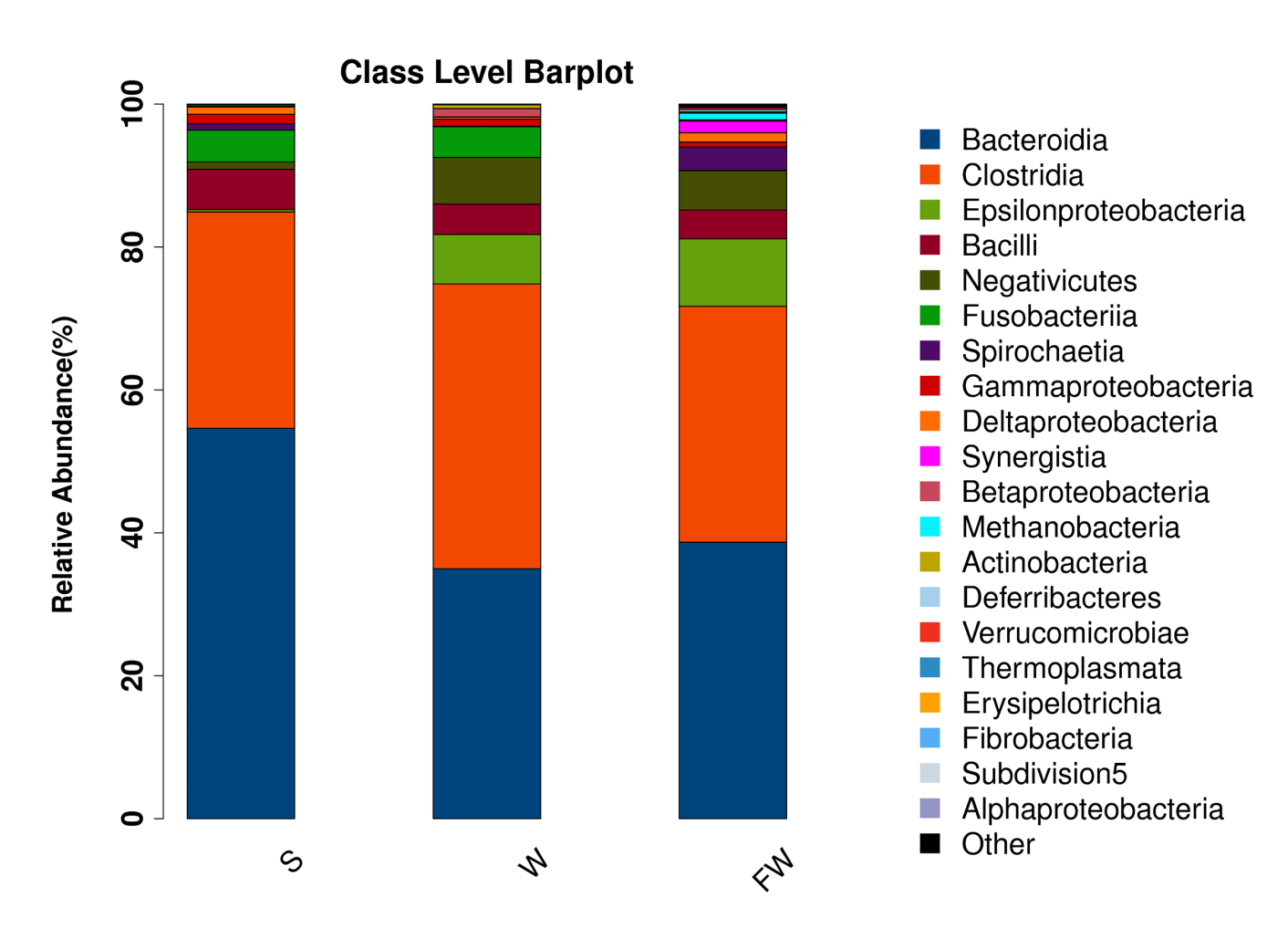


**Supplementary Figure 1.** The class composition of colonic microbiomeamong three groups.


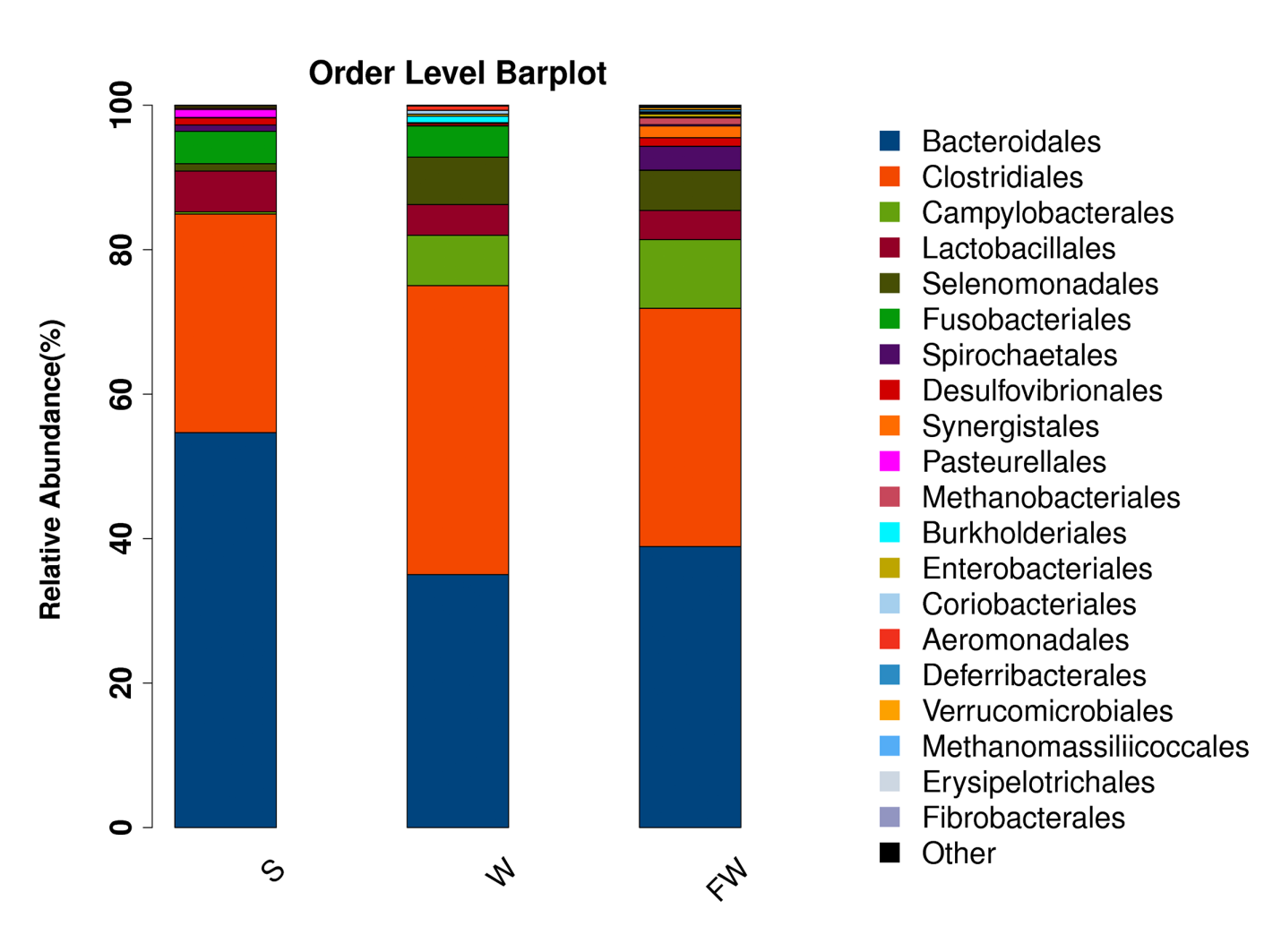
**Supplementary Figure 2.** The order composition of colonic microbiome in nursing and weaned piglets.

## Supplementary Tables

**SupplementaryTable1.** Ingredient composition and nutritional levels of basic diet

| Item |  | Nutritional level^2^ |  |
| --- | --- | --- | --- |
| Corn | 567 | Digestible Energy (MJ/kg) | 13.98 |
| Puffed soybean | 130 | Crude protein (g/Kg) | 191 |
| Soybean meal | 155 | Crude fat (g/Kg) | 65.7 |
| Sucrose | 10 | Ash (g/Kg) | 53.1 |
| Fish meal | 30 | Moisture (g/Kg) | 110.3 |
| Whey powde | 30 | Calcium (g/Kg) | 9.3 |
| Plasma protein powder | 10 | Phosphorus (g/Kg) | 6.5 |
| Soybean oil | 10 | Lysine (g/Kg) | 11.5 |
| Stone powder | 8 | Methionine (g/Kg) | 3.0 |
| Calcium dihydrogen phosphate | 10 |  |  |
| Vitamin premix^1^ | 40 |  |  |

^1^Provided per kilogram of diet: 16,000 IU vitamin A, 4000IU vitamin D_3_, 100 IU vitamin E, 0.5mg vitamin K_3_, 2mg vitamin B_1_, 4.5mg vitamin B_2_, 7mg vitamin B_6_, 0.03mg vitamin B_12_, 0.2mg biotin, 10mg folic acid, 30mg nicotinic acid, 22mg pantothenic acid; 85mg Fe(FeSO_4_), 100mg Cu(CuSO_4_),0.3mg Mn(MnSO_4_), 0.14 mg I(CaI_2_).

^2^The data regarding crude protein, crude fat, crude ash, moisture, calcium and total phosphorus are measured values, the others are calculated values.

**SupplementaryTable2.** Target genes detected in the study and their primers sequences

| Gene | Genbank accession | Primer sequence (5’- 3’) | Size(bp) | Annealing (℃) |
| --- | --- | --- | --- | --- |
| IL-6 | NM_214399.1 | GCCTTCAGTCCAGTCGCCTTCT | 101 | 60 |
|  |  | GTGGCATCACCTTTGGCATCTTC |  |  |
| IL-10 | NM_214041.1 | GACCAGATGGGCGACTTGTTG | 160 | 60 |
|  |  | GGGAGTTCACGTGCTCCTTGAT |  |  |
| TNF-α | NM_001143690.1 | CGCTCTTCTGCCTACTGCACTT | 156 | 60 |
|  |  | CGGCTTTGACATTGGCTACAA |  |  |

| tax_name | S | W | FW |
| --- | --- | --- | --- |
| *Bacteroidetes* | 55.75 | 35.01 | 41.70 |
| *Firmicutes* | 35.09 | 50.24 | 40.03 |
| *Proteobacteria* | 3.28 | 9.31 | 10.85 |
| *Fusobacteria* | 4.37 | 4.30 | 0.03 |
| *Spirochaetes* | 0.79 | 0.06 | 2.98 |
| *Synergistetes* | 0.05 | 0.01 | 1.52 |
| *Euryarchaeota* | 0.16 | 0.01 | 1.02 |
| *Actinobacteria* | 0.07 | 0.53 | 0.15 |
| *Verrucomicrobia* | 0.00 | 0.00 | 0.35 |
| *Deferribacteres* | 0.00 | 0.00 | 0.27 |
| *Fibrobacteres* | 0.00 | 0.00 | 0.12 |
| *CandidatusSaccharibacteria* | 0.01 | 0.00 | 0.00 |
| *Elusimicrobia* | 0.00 | 0.00 | 0.01 |
| *Planctomycetes* | 0.00 | 0.00 | 0.00 |
| *Acidobacteria* | 0.00 | 0.00 | 0.00 |

**SupplementaryTable3.** The phyla composition of colonic microbiome among three groups

**SupplementaryTable4.** The class composition of colonic microbiome among three groups

| tax_name | S | W | FW |
| --- | --- | --- | --- |
| *Bacteroidia* | 50.51 | 34.73 | 35.79 |
| *Clostridia* | 27.46 | 39.45 | 30.63 |
| *Epsilonproteobacteria* | 0.28 | 6.86 | 8.42 |
| *Bacilli* | 5.23 | 4.19 | 3.75 |
| *Negativicutes* | 0.95 | 6.48 | 5.05 |
| *Fusobacteriia* | 4.37 | 4.30 | 0.03 |
| *Spirochaetia* | 0.79 | 0.06 | 2.98 |
| *Gammaproteobacteria* | 1.29 | 0.99 | 0.65 |
| *Deltaproteobacteria* | 0.92 | 0.29 | 1.19 |
| *Synergistia* | 0.05 | 0.01 | 1.52 |
| *Betaproteobacteria* | 0.08 | 1.17 | 0.15 |
| *Methanobacteria* | 0.15 | 0.01 | 0.88 |
| *Actinobacteria* | 0.07 | 0.53 | 0.15 |
| *Deferribacteres* | 0.00 | 0.00 | 0.27 |
| *Verrucomicrobiae* | 0.00 | 0.00 | 0.24 |
| *Thermoplasmata* | 0.01 | 0.00 | 0.14 |
| *Erysipelotrichia* | 0.01 | 0.08 | 0.04 |
| *Fibrobacteria* | 0.00 | 0.00 | 0.12 |
| *Subdivision5* | 0.00 | 0.00 | 0.10 |
| *Alphaproteobacteria* | 0.01 | 0.00 | 0.03 |
| *Other* | 7.84 | 0.85 | 7.86 |

**SupplementaryTable5.** The order composition of colonic microbiome among three groups

| tax_name | S | W | FW |
| --- | --- | --- | --- |
| *Bacteroidales* | 50.51 | 34.73 | 35.79 |
| *Clostridiales* | 27.38 | 39.45 | 30.44 |
| *Campylobacterales* | 0.28 | 6.86 | 8.42 |
| *Lactobacillales* | 5.23 | 4.19 | 3.75 |
| *Selenomonadales* | 0.95 | 6.48 | 5.05 |
| *Fusobacteriales* | 4.37 | 4.30 | 0.03 |
| *Spirochaetales* | 0.79 | 0.06 | 2.98 |
| *Desulfovibrionales* | 0.91 | 0.29 | 1.07 |
| *Synergistales* | 0.05 | 0.01 | 1.52 |
| *Pasteurellales* | 1.06 | 0.10 | 0.15 |
| *Burkholderiales* | 0.07 | 0.88 | 0.10 |
| *Methanobacteriales* | 0.15 | 0.01 | 0.88 |
| *Enterobacteriales* | 0.21 | 0.29 | 0.38 |
| *Coriobacteriales* | 0.05 | 0.52 | 0.15 |
| *Aeromonadales* | 0.01 | 0.59 | 0.12 |
| *Deferribacterales* | 0.00 | 0.00 | 0.27 |
| *Verrucomicrobiales* | 0.00 | 0.00 | 0.24 |
| *Methanomassiliicoccales* | 0.01 | 0.00 | 0.14 |
| *Erysipelotrichales* | 0.01 | 0.08 | 0.04 |
| *Fibrobacterales* | 0.00 | 0.00 | 0.12 |
| *Other* | 7.96 | 1.16 | 8.35 |

**SupplementaryTable6.** The family composition of colonic microbiome among three groups

| tax_name | S | W | FW |
| --- | --- | --- | --- |
| *Prevotellaceae* | 32.94 | 20.42 | 18.58 |
| *Ruminococcaceae* | 16.93 | 18.95 | 15.16 |
| *Lachnospiraceae* | 6.45 | 19.52 | 10.09 |
| *Porphyromonadaceae* | 12.72 | 4.03 | 10.77 |
| *Bacteroidaceae* | 3.84 | 10.15 | 4.79 |
| *Campylobacteraceae* | 0.27 | 6.83 | 7.75 |
| *Lactobacillaceae* | 5.12 | 4.13 | 3.74 |
| *Fusobacteriaceae* | 4.37 | 4.30 | 0.03 |
| *Veillonellaceae* | 0.30 | 5.55 | 0.79 |
| *Acidaminococcaceae* | 0.65 | 0.94 | 4.26 |
| *Spirochaetaceae* | 0.79 | 0.06 | 2.98 |
| *Desulfovibrionaceae* | 0.91 | 0.29 | 1.05 |
| *Rikenellaceae* | 0.66 | 0.03 | 1.12 |
| *Synergistaceae* | 0.05 | 0.01 | 1.52 |
| *Pasteurellaceae* | 1.06 | 0.10 | 0.15 |
| *Methanobacteriaceae* | 0.15 | 0.01 | 0.88 |
| *Enterobacteriaceae* | 0.21 | 0.29 | 0.38 |
| *Sutterellaceae* | 0.04 | 0.76 | 0.05 |
| *Coriobacteriaceae* | 0.05 | 0.52 | 0.15 |
| *Succinivibrionaceae* | 0.01 | 0.59 | 0.12 |
| *Other* | 12.48 | 2.54 | 15.65 |

**SupplementaryTable7.** The genus composition of colonic microbiome among three groups

| tax_name | S | W | FW |
| --- | --- | --- | --- |
| *Prevotella* | 19.63 | 16.61 | 7.04 |
| *Alloprevotella* | 7.88 | 1.39 | 9.78 |
| *Bacteroides* | 3.84 | 10.15 | 4.79 |
| *Campylobacter* | 0.27 | 6.83 | 7.75 |
| *Lactobacillus* | 5.12 | 4.13 | 3.74 |
| *Fusobacterium* | 4.28 | 4.30 | 0.03 |
| *Barnesiella* | 4.94 | 0.97 | 1.44 |
| *Roseburia* | 1.16 | 3.89 | 1.55 |
| *Faecalibacterium* | 0.04 | 6.23 | 0.24 |
| *Phascolarctobacterium* | 0.65 | 0.94 | 4.13 |
| *Oscillibacter* | 3.00 | 0.51 | 1.49 |
| *Clostridium XlVb* | 1.10 | 1.77 | 0.59 |
| *Parabacteroides* | 1.44 | 0.27 | 1.37 |
| *Megasphaera* | 0.04 | 2.64 | 0.31 |
| *Clostridium XlVa* | 0.47 | 1.62 | 0.84 |
| *Butyricicoccus* | 0.04 | 1.69 | 0.90 |
| *Treponema* | 0.58 | 0.02 | 1.95 |
| *Blautia* | 0.05 | 2.29 | 0.20 |
| *Flavonifractor* | 0.00 | 2.00 | 0.02 |
| *Gemmiger* | 0.33 | 1.48 | 0.18 |
| *Other* | 45.15 | 30.25 | 51.67 |
